# Recent changes in growth trajectories: a population-based cohort study of over 5 million Brazilian children born between 2001 and 2014

**DOI:** 10.1016/j.lana.2024.100721

**Published:** 2024-03-27

**Authors:** Carolina Santiago-Vieira, Gustavo Velasquez-Melendez, Rita de Cássia Ribeiro-Silva, Elizabete de Jesus Pinto, Maurício L. Barreto, Leah Li

**Affiliations:** a School of Nursing, Universidade Federal de Minas Gerais, Belo Horizonte, Brazil; b School of Nutrition, Federal University of Bahia, Salvador, Brazil; c Center of Data and Knowledge Integration for Health (CIDACS), Fiocruz Bahia, Salvador, Brazil; d Health Sciences Center, Federal University of Recôncavo da Bahia, Santo Antônio de Jesus, Brazil; e Population, Policy and Practice Research and Teaching Department, Great Ormond Street Institute of Child Health, University College London, London, UK

**Keywords:** Body mass index, Height, Growth trajectories, Changes, Brazilian children

## Abstract

**Background:**

There is limited evidence on recent trends in childhood growth trajectories in Low-/middle-income countries. We investigated how age-trajectories for height and Body Mass Index (BMI) have changed among Brazilian children born in two different time periods after 2000.

**Methods:**

We used a population-based cohort (part of the “Cohort of 100-Million Brazilians”) created by the linkage of three Brazilian administrative databases: the Cadastro Único of the Federal Government, the National System of Live Births and the National Nutritional and Food Surveillance System. We included longitudinal data on 5,750,214 children who were 3 to <10 years of age and born between 2001 and 2014 (20,209,133 observations). We applied fractional polynomial models with random-effects to estimate mean height and BMI trajectories for children.

**Findings:**

Compared to children born in 2001–2007, the cohort born in 2008–2014 were on average taller, by a z-score of 0.15 in boys and 0.12 in girls. Their height trajectories shifted upwards, by approximately 1 cm in both sexes. Levels of BMI increased little, by a z-score of 0.06 (boys) and 0.04 (girls). Mean BMI trajectories also changed little. However, the prevalence of overweight/obesity increased between cohorts, e.g., from 26.8% to 30% in boys and 23.9%–26.6% in girls aged between 5 and <10 years.

**Interpretation:**

An increase of 1 cm in mean height of Brazilian children during a short period indicates the improvement in maternal and child health, especially those from low-income families due to the new health and welfare policies in Brazil. Although mean BMI changed little, the prevalence of child overweight/obesity slightly increased and remained high.

**Funding:**

This work was supported by 10.13039/501100003593National Council for Scientific and Technological Development – CNPq; Coordenação de Aperfeiçoamento de Pessoal de Nível Superior – CAPES; 10.13039/501100000272National Institute for Health Research (NIHR) Great Ormond Street Hospital Biomedical Research Centre; 10.13039/100012686Society for the Study of Human Biology; Fundação de Amparo à Pesquisa do Estado de Minas Gerais – FAPEMIG; Departamento de Ciência e Tecnologia da Secretaria de Ciência, Tecnologia, Inovação e Complexo da Saúde do Ministério da Saúde - Decit/SECTICS/MS. The study also used resources from the Centre for Data and Knowledge Integration for Health (CIDACS), which receives funding from the 10.13039/100000865Bill & Melinda Gates Foundation, the 10.13039/100010269Wellcome Trust, the Health Surveillance Secretariat of the 10.13039/100009647Ministry of Health and the 10.13039/501100010253Secretariat of Science and Technology of the State of Bahia (SECTI-BA).

**Research in context**

**Evidence before this study**

Poor growth in height, and high BMI, are two markers of the double burden of malnutrition in children, and both have been linked with poorer cardiometabolic risk in adulthood. We searched for studies, including reviews, of secular trends in child growth published from Jan 1990 to April 2023. We searched in PubMed, Embase and Web of Science with the “AND” logic combination for ‘childhood’, ‘trends’ and ‘height/BMI’ concepts with the following standard searching strategy. For ‘**childhood’**, MeSH terms: “child”, “children”, free text terms: “child∗”. For ‘**trends**’, MeSH terms: “changes”, “trends”. Free-text terms: “change∗”, “increase∗”, “decrease∗”, “trends∗”, “secular∗”, “persist∗”, “unchanged∗∗”. For ‘**height/BMI**’, MeSH terms: “obesity”, “overweight”, “adiposity”, “body mass index”, “body weight”, “body height”, “body size”. Free-text terms: “obes∗”, “overweight”, “adiposity”, “BMI”, “body mass index”, “body weight”, “body height”, “stature”, “body size”. We identified 2002 papers (actual papers would be fewer when removing duplicates or those do not satisfy the inclusion criteria). Studies consistently show that height of children have increased during the 20th century due to the improvement in child nutrition and health. But the trends varied across the globe and differed between countries with different economic levels. In most populations, overweight and obesity continue to increase in recent decades and across all age groups. Globally, the prevalence of obesity in children increased rapidly both in boys and girls, but trends slowed down in some populations, especially in High Income Countries, whereas findings from Low-middle Income Countries are mixed. Evidence is limited for changes in age-trajectories for height and body sizes.

**Added value of this study**

This study adds new information by estimating changes in growth trajectories for height and BMI in recent years using longitudinal growth data on over 5 million children born in recent years from “Cohort of 100-Million Brazilians”. Our analysis shows that height of Brazilian children increased by 1 cm on average during a short period (2001–2007 to 2008–2014). The prevalence of child overweight remained high with a small increase, although mean BMI changed little during the same period.

**Implications of all the available evidence**

Our results reveal an increase in height and overweight in Brazilian children. These patterns are consistent with findings in many LMICs. These findings reinforce the urgent need to develop strategies for interventions early in life to prevent the development of obesity especially children from low-income families.

## Introduction

It is well recognised that growth and development at different stages in childhood are associated with adult chronic diseases.^1^ In particular, short stature, an important biomarker of poor early life environment and nutrition, is associated with adult mortality.^2^ On the other hand, increased body mass index (BMI) has an important influence on a range of adverse health outcomes, notably cardiovascular diseases, diabetes and some forms of cancers.^3^

Height increased during the 20th century due to the improvement in child nutrition and health. But the trends varied across countries with different economic levels.^4^ A study based on data from 200 countries showed that the mean height of school-aged children and adolescents increased between 1985 and 2019 in most countries.^5^ The increasing trend was slower in many high-income countries (HICs) compared to low- and middle-income countries (LMICs), especially some emerging economies.^5^ In a study conducted in nine cities from northern, central and southern regions of China, mean height of children under 7 years increased by about 3.5 cm between 1975 and 2015.^6^

In most populations, overweight and obesity continue to increase among all age groups.^7^^,^^8^ Globally, the prevalence of obesity increased rapidly, from 0.7% in 1975 to 5.6% in 2016 in girls aged 5–19 years, and from 0.9% to 7.8% respectively in boys.^7^ More recently, the global prevalence of overweight among children under 5 years increased slightly, from 5.4% (33 million) in 2002 to 5.7% (38.9 million) in 2020.^9^ In some HICs, the trends for overweight and obesity are becoming stable, although the prevalence remains high,^7^ e.g., at around 7.8% for overweight for under 5 years in 2020.^9^ In many LMICs, mean BMI and prevalence of overweight/obesity continue to increase.^7^^,^^8^

In Brazil, the mean height of children has increased since the 1950s,^10^ whereas the trends for increases in overweight/obesity or mean BMI are less consistent. For children under 5 years, the prevalence of overweight/obesity was stable between 1974-1975 and 2006–2007,^11^ at 6–7% in a nationally representative sample.^12^ A small increase from 11.6% in 2009 to 12.6% in 2017 was found from the Food and Nutrition Surveillance System in Brazil.^13^

There is limited evidence on recent trends in growth trajectories of children in LMICs, due to the scarcity of longitudinal growth data collected on cohorts from the same population but at different time periods. The upward shift of height and BMI trajectories has been found in recent years among Chinese children.^14^ To our knowledge, there is no study that has reported recent changes in growth trajectories in Brazilian children. The new health and welfare policies in Brazil would have impacted on nutritional status of children, and their growth trajectories. These changes are likely to have implications for morbidity and mortality in future adults.^2^^,^^15^ Thus, it is important to understand how height and BMI trajectories have changed in recent years.^16^ Furthermore, there is evidence suggesting that the increase obesity in Brazil among the low-income population is occurring at a faster rate compared to what is observed in the high-income population.^17^ Using the longitudinal data from a large population cohort of over 5 million Brazilian children from poor economic background in Brazil, we aimed to investigate the extent to which growth trajectories for height and BMI have changed in recent years.

## Methods

The population-based cohort was part of the “Cohort of 100 Million Brazilians”, which was created by the linkage of three Brazilian administrative databases: the *Cadastro Único* (CadUnico), the Live Birth Information System (SINASC) and the Food and Nutrition Surveillance System (SISVAN). The linked data are available from The Centre for Data and Knowledge Integration for Health (CIDACS)/Fiocruz, Brazil.^18^

The CadUnico is a shared registry for more than 20 social programs and consists of 114,008,317 adults and children registered between January 2008 and December 2017 who lived in poverty, i.e., from families with a monthly income ≤3 times of minimum wage (∼US$750). In 2015, it included >50% of Brazilian population.^18^ This database contains demographic and socio-economic data.^19^ Each individual registered in the CadUnico was given a unique non-transferable personal identification number (NIS).

The SINASC contains detailed information on mothers and newborns throughout Brazil, including characteristics of the mother and newborn, prenatal care, obstetric history, delivery, and birth.^20^ SINASC is a valuable and reliable instrument,^21^ with 97.9% completeness in the variables recorded at hospital deliveries and coverage of more than 97% of live births in Brazil.^22^

The SISVAN is an important system for monitoring the food and nutritional situation of the population, structurally linked to primary care in Brazil, at all stages of life.^23^ For children under 10 years of age, sex, date of birth, weight and height are recorded.^24^ The frequency of data collection follows the recommendation from the Ministry of Health (at least 9 follow-ups during the first two years and annually thereafter).^25^ The assessment includes anthropometric and food consumption data between 2008 and 2017 with 307,245,508 records (from 59,724,164 individuals).

Three data sources were linked as following: first, the CadUnico (cohort baseline) was linked to SINASC through non-deterministic linkage based on key attributes (name, sex, children’s date of birth, mother's name, and the municipality of residence).^26^^,^^27^ Second, the cohort baseline was linked to SISVAN through a deterministic linkage using the NIS. For individuals without the NIS, non-deterministic linkage was performed using common attributes (name, mother's name, date of birth and sex). The cohort was resulted from the merge of three databases. Our study focused on children from age three to under ten years. The CIDACS-RL (96% accuracy) binding tools were developed and used for the data linkage.^28^ CIDACS-RL was developed with the main objective of linking large data sets in CIDACS, and uses a combination of indexing algorithms to find the most similar records between the two databases, allowing pairwise comparison instead of limiting the comparison with a common blocking step.^28^

As the baseline cohort (CadUnico) included children from relatively low-income families, the study sample is not representative of Brazilian children in the population.

The present study was approved by the research ethics committee of the Federal University of Minas Gerais (reference number 37534620.3.0000.5149).

### Study population

**Fig. 1:**
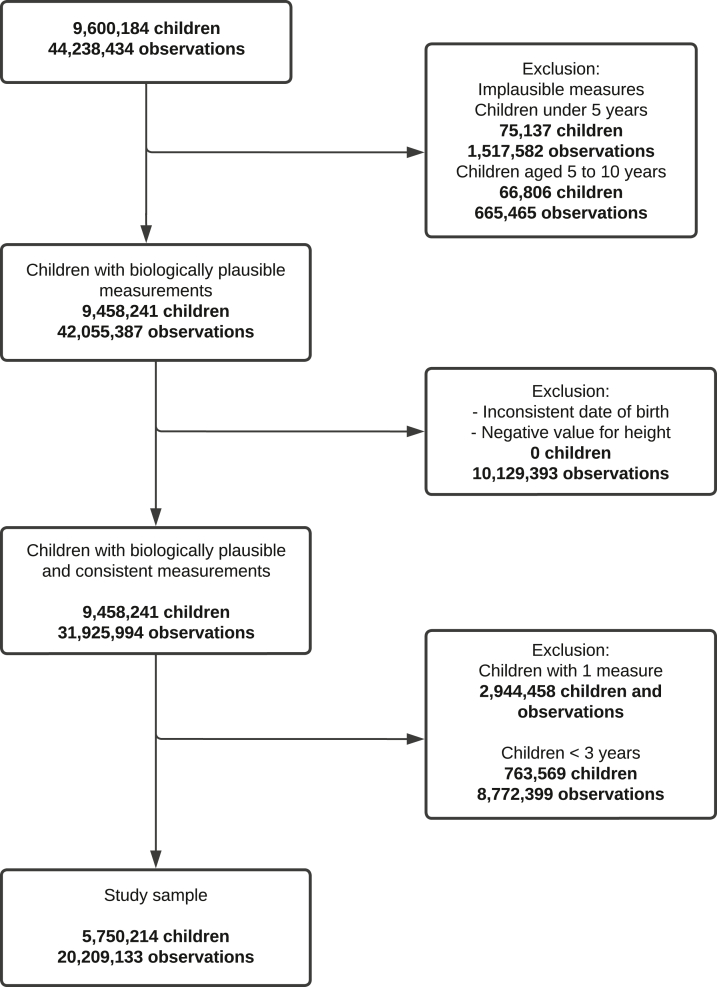
**Flowchart for selection of the study sample (children aged from 3 to under 10 years with repeated height and/or BMI measures between 2008 and 2017)**.

The study sample included 5,750,214 children (2,770,805 boys and 2,979,409 girls) aged from 3 to under 10 years with repeated (≥2) height and/or BMI measures between 2008 and 2017 (total 20,209,133 measurements). Children with missing age, sex, weight or height measure, or with implausible values according the WHO cutoffs for age- and sex-standardized BMI z-scores (<−5 or >5), height z-scores (<−6 or >6) and weight z-scores (<−6 or >5) were excluded.^29^^,^^30^ We also excluded children with inconsistent date of birth or a negative value for height change across follow-ups. A detailed flowchart about the section of the study sample is provided in Fig. 1. We derived two birth cohorts who were born between 2001 and 2007 (N = 1,955,050) and between 2008 and 2014 (N = 3,795,164).

### Measures

Birthweight of each child was recorded in the SINASC. Height (to nearest 0.1 cm) and weight (0.1 kg) were measured at each follow-up in the SISVAN in light clothes without shoes.^24^ BMI (kg/m2) was calculated. We derived age- and sex-standardized z-scores for height and BMI using WHO growth reference for under 5 years^29^ and 5–10 years.^30^ For children under 5 years, overweight and obesity was defined as weight for height z-scores above two and three standard deviations respectively. The respective cut-offs for children aged 5–10 years were one and two standard deviations.^31^

### Statistical analysis

All analyses were stratified by sex. As height and BMI measurements (level-1) were clustered within children (level-2 units), we first applied mixed effects linear models to estimate mean birthweight, mean z-scores for height and BMI for each cohort, and changes (and 95% confidence intervals) between two cohorts. To establish the extent to which the age-trajectories for height and BMI in childhood have changed in recent years, we applied fractional polynomial models with random effects to estimate the mean age-trajectories for height and BMI. We explored fractional polynomial functions of age to capture the nonlinear curves for growth trajectories for each cohort separately. Models were selected based on the Akaike information criterion (AIC), Bayesian information criterion (BIC), and likelihood ratio test. The best-fitting two-degree fractional polynomials were the same for both cohorts, and for height trajectories included *age* and *age*^3^ for both sexes. For BMI trajectories, they included *age* and *age*^2^ for boys, and *age* and *age∗* log(*age*) for girls. These models allow children with different number and timing of measurements. The observed and estimated mean height and BMI trajectories were presented in Supplementary Figs. S1 and S2, respectively. To illustrate our findings, we plotted the mean height and BMI trajectories for each cohort (born in 2001–2007 and 2008–2014). All analyses were conducted in Stata (Version 15.1).

### Role of funding source

We would like to declare that the funders of this study had no involvement whatsoever in the study design, data analysis, interpretation of data, or writing of the manuscript. The research conducted herein was solely the responsibility of the authors.

## Results

**Fig. 2:**
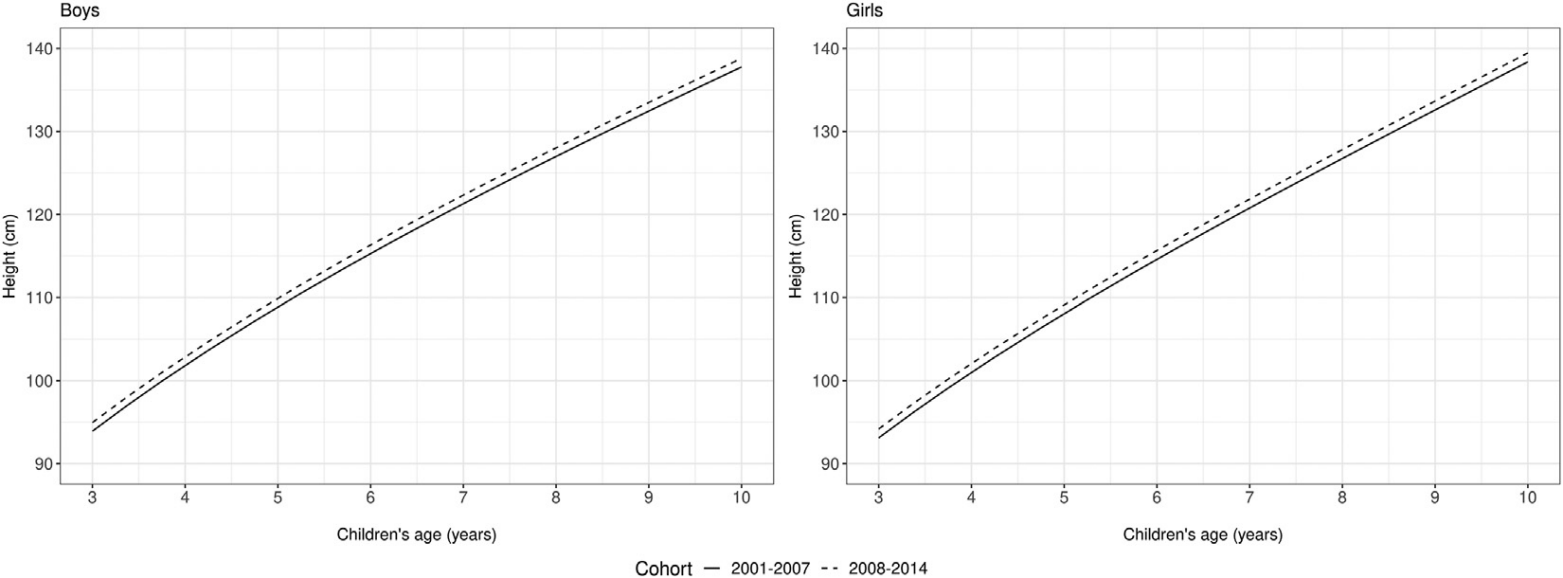
**Mean trajectories of height (cm) from 3 to 10 years for boys and girls in two cohorts born in 2001–2007 and 2008–2014 (N = 5,750,214).** The mean trajectory for the two cohorts was estimated using mixed effects models with fractional polynomials†. Legend: †Solid line is for the cohort born in 2001–2007 and dotted line is for the cohort born in 2008–2014.

The 5,750,214 children included in the analyses had an average number of 3.5 measures per child. Compared to children born in 2001–2007, the cohort born in 2008–2014 had a small increase in mean birthweight (by ∼30 g) and were taller by a height z-score of 0.145 (0.143, 0.147) in boys and 0.119 (0.117, 0.122) in girls (Table 1). The mean trajectories for height (age 3–10 years) shifted upwards between cohorts, with boys and girls born in 2008–2014 being taller than those born in 2001–2007 (Fig. 2) by approximately 1 cm across ages for both sexes (Supplementary Table S1).

**Table 1:** Mean birthweight (g), BMI and height z-scores^a^, overweight, obesity, maternal education, residence area, residence region and changes between boys and girls born in 2001–2007 and 2008–2014.

	Cohort 1 (2001–2007)	Cohort 2 (2008–2014)	Change (95% CI)
	N = 1,955,050	N = 3,795,164	
Boys			
Birthweight (g)	3178.7	3208.5	29.8 (28.5, 31.1)
Height for age (z-score)	−0.194	−0.339	0.14 (0.14, 0.15)
BMI for age (z-score)	0.208	0.268	0.06 (0.05, 0.06)
Overweight/obesity (3–4 years)	10.86%	11.83%	0.97 (0.95, 0.99)
Obesity	4.03%	4.52%	0.49 (0.48, 0.50)
Overweight/obesity (5–10 years)	26.83%	30.00%	3.17 (3.14, 3.20)
Obesity	11.09%	13.81%	2.72 (2.70, 2.74)
Girls			
Birthweight (g)	3062.0	3091.4	29.4 (28.2, 30.6)
Height for age (z-score)	−0.148	−0.267	0.12 (0.11, 0.12)
BMI for age (z-score)	0.112	0.155	0.04 (0.04, 0.05)
Overweight/obesity (3–4 years)	9.60%	10.46%	0.86 (0.84, 0.88)
Obesity	3.58%	3.86%	0.28 (0.27, 0.29)
Overweight/obesity (5–10 years)	23.94%	26.64%	2.70 (2.67, 2.72)
Obesity	9.13%	11.22%	2.09 (2.07, 2.10)
Maternal education			
None	3.75%	1.83%	−1.92 (−1.93, −1.91)
1–3 years	17.39%	9.53%	−7.86 (−7.88, −7.83)
4–7 years	45.28%	36.95%	−8.33 (−8.35, −8.30)
≥8 years	33.59%	51.68%	18.09 (18.06, 18.12)
Residence area			
Urban	67.80%	72.28%	4.48 (4.45, 4.51)
Rural	32.20%	27.72%	−4.48 (−4.51, −4.45)
Residence region			
North	11.35%	14.61%	3.26 (3.24, 3.28)
Northeast	44.69%	44.53%	−0.16 (−0.19, −0.13)
Southeast	27.01%	25.93%	−1.08 (−1.11, −1.05)
South	11.36%	8.93%	−2.43 (−2.45, −2.41)
Midwest	5.59%	6.00%	0.41 (0.39, 0.42)

a Age- and sex-standardized z-scores according to the WHO reference.^29^^,^^30^

**Fig. 3:**
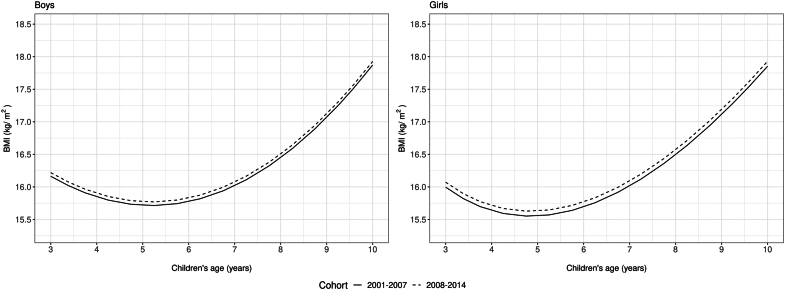
**Mean trajectories of BMI (kg/m2) from aged 3 to 10 years for boys and girls in two cohorts born in 2001–2007 and 2008–2014 (N = 5,750,214).** The mean trajectory for the two cohorts was estimated using mixed effects models with fractional polynomials†. Legend: †Solid line is for the cohort born in 2001–2007 and dotted line is for the cohort born in 2008–2014.

There was a slight increase in mean BMI z-scores between cohorts: by 0.060 (0.057, 0.063) in boys and 0.043 (0.040, 0.046) in girls (Table 1). Mean BMI trajectories changed little (Fig. 3), with only small increases in mean BMI of ∼0.06 kg/m^2^ (boys) and ∼0.08 kg/m^2^ (girls) (Supplementary Table S2). However, the prevalence of overweight/obesity increased between cohorts (2001–2007 to 2008–2014), e.g., from 10.9% to 11.8% for boys and 9.6% to 10.5% for girls under 5 years, and from 26.8% to 30% and 23.9% to26.6% respectively for boys and girls between aged 5 years and under 10 years (Table 1).

## Discussion

Using the longitudinal growth data from a population cohort of 5.75 million Brazilian children aged 3 to under 10 years, we found an upward shift of height trajectories by 1 cm between boys and girls born in 2001–2007 and those born in 2008–2014. There was a modest increase in birthweight. Although BMI growth trajectories changed little, the prevalence of overweight/obesity remained high, especially for children 5 years or over, and increased during the short period (from 24–27% to 27–30%).

### Changes in height trajectories

Trends of increasing height have been reported in many populations, including emerging countries such as China, South Korea, Southeast Asia, the Middle East, North Africa, Latin America, and the Caribbean.^5^ A stagnation or decrease in mean height has been found in countries from sub-Saharan Africa in children aged 5–19 years between 1971 and 2019.^5^ While trends in childhood growth are well documented,^32^^,^^33^ the evidence on changes in growth trajectories over time is less explored. Few studies have reported recent trends for height trajectories. One study of four longitudinal British birth cohorts born between 1946 and 2000/1 showed increases in child-to-adolescent trajectories (7–15 years) for height between cohorts born in 1970 or earlier and those born in 2000/1.^34^ In LMICs, a recent study using data from the China Health and Nutrition Survey shows that height trajectories (7–18 years) have shifted upwards from children born in 1981–1985 to those born in 1996–2000.^14^

There is a trend of increasing height in Brazil, by 1 cm from 1952 to 1967 and by 2.4 cm from 1967 to 1982 among boys and girls in five Brazilian macro-regions.^10^ National studies that have evaluated the recent trends in height (i.e., after the 2000s) are scarce, and none has investigated recent changes in height trajectories of Brazilian children.

We found that height trajectories from age 3–10 years shifted upward by 1 cm between Brazilian children born in 2001–2007 and 2008–2014. Taller stature is associated with better health outcomes, such as a lower likelihood of heart disease and stroke and greater longevity.^12^ The increase of 1 cm in height of Brazilian children over such a short period of time reflects the rapid economic development in recent years,^35^ and improvement of living standards of children from low-income families. In our study population, maternal education has improved between the cohort born in 2001–2007 and 2008–2014 (e.g., proportion of mothers had ≥8 years schooling increased from 34% to 52%) and more children are living in the urban region, from 67.8% to 72.3%. In Brazil, there has been improvement in social, sanitary and health conditions, such as reduction of poverty and fertility, increase in maternal schooling, expansion of urbanization, improvement in access to potable water and sanitation.^12^

The implementation of national policies and programs in 1990–2015 after the creation of the Unified Health System (SUS) in 1988 to improve maternal and child health care services^12^^,^^36^ have contributed to the improvement in child health in Brazil. For example, infant mortality has decreased substantially between 1990 and 2015, from 53.7 to around 15.6 per 1000 live births, meeting the target 4 of the millennium goals.^36^ Importantly, the conditional cash transfer program for families in situations of social vulnerability, the Bolsa Família Program (PBF), contributed substantially to breaking the intergenerational cycle of poverty after 2003.^37^ It has been reported that the program was responsible for a 17% reduction in infant mortality rate.^38^ These improvements have been reflected in the trends of increasing height in Brazilian children in recent years reported in our study.

### Changes in BMI trajectories and prevalence of overweight/obesity

Trends in mean BMI or the prevalence of overweight/obesity have been well studied worldwide. A study using pooled data from 200 countries showed an increase in mean BMI in children and adolescents in almost all countries before the year 2000. After 2000, the evidence suggests that the trend of increasing childhood BMI has flattened in HICs, but continues in some LMICs (e.g., parts of Asia).^7^^,^^39^

However, few studies explored how age-trajectories for childhood BMI have changed in recent years. In the China Health and Nutrition Survey, BMI trajectories shifted upwards between children born in 1981–1985 and 1996–2000, and the trends were smaller in BMI than in height.^14^ In Brazil, studies have shown that the direction of the trend has changed over time. The prevalence of overweight was stable between 1974 and 2006^12^ and increased between 2009 and 2017.^13^ In our study, although we found little increase in BMI trajectories among children born after 2000, the prevalence of overweight and obesity remained high and further increased during a short period, especially for children aged 5–10. Given the current high prevalence of overweight and obesity, it is important to continue monitoring Brazilian children’s growth.

In recent decades, Brazil has experienced a rapid nutritional, demographic and epidemiological transition.^40^ In LMICs including Brazil, dietary patterns have changed, such as increased consumption of ultra-processed foods, rich in carbohydrates, saturated fat, sugar and salt.^41^ There is also a marked increase in physical inactivity and sedentary behavior.^42^ These changes were likely to have contributed to the increased risk of overweight and obesity-related diseases.^43^

Children from low-income families face greater restrictions in access to recreation and physical activity. The lack of safety in some urban areas can inhibit children’s outdoor activities, worrying parents about their children’s safety.^44^ Poverty and violence in neighbourhoods are also important environmental factors that impact children's outdoor physical activity.^45^ Research has shown that children who spend more time outdoors accumulate more time for physical activity,^46^ are more active, less sedentary, and are less likely to face difficulties in relationships with their peers,^47^ and, in addition, children who spend more time playing outdoors have lower BMI scores.^48^

The Brazilian government has implemented public policies for promoting healthy eating. These policies are established through the Brazilian guide for healthy eating for all life cycles and are rooted in the principles of proper and nutritious nutrition. They serve as a means of facilitating food and nutritional education initiatives within the Unified Health System.^49^ The importance of policies and programs that promote healthier food choices and nutritional education for children and their families, promote physical activity from childhood and creation of safe environments for outdoor physical activities, help to combat sedentary lifestyle and promote a healthier lifestyle, is highlighted.

While height growth of Brazilian children has improved, the recent pandemic may have adversely affected family income and nutritional status, which may result in a slowdown in secular trend in height growth of children, especially those from low-income families. More sedentary lifestyle due to school closures and restrictions on leaving the house may have also led to an increase in child obesity, as reported in some populations.^50^ Future studies extend to recent data from 2020 will provide important evidence on the impact of pandemic on child growth and health.

### Strengths and limitations

To our knowledge, this is the first study to use longitudinal growth measures from such a large population (e.g., more than 5 million children) to investigate recent changes in growth trajectories. Mixed-effects models applied here accounted for within individuals’ correlations and allowed the inclusion of children with different numbers and timing of measurements. However, limitations exist. While the sample was selected across Brazil, our study included children predominantly from low-income families. Therefore, the findings should be interpreted with caution and not be generalized to the Brazilian child population. Nevertheless, the trends in child growth reflect the impact of health and welfare politics, and the future government strategy and interventions should continue to target this population of children the most.

### Conclusions

Mean height of Brazilian children increased 1 cm during a short period, indicating the improvement of maternal and child health due to Brazil's new health and welfare policies. The prevalence of child overweight/obesity remained high and slightly increased, even though mean BMI little changed. Therefore, there is a need to develop strategies for interventions early in life to prevent the development of obesity. Early interventions can be effective, especially for children from disadvantaged backgrounds, in preventing the development of obesity and chronic diseases in later life.

## Contributors

CSV, LL and GVM designed the study. CSV and LL contributed to the data analysis and wrote the manuscript. RCRS, EJP, MLB and GVM provided critical feedback regarding the analyses and the manuscript. CSV, LL and GVM are the guarantors of this work. All authors approved the final manuscript and accept responsibility for the decision to submit for publication.

## Data sharing statement

All data supporting this study were obtained from the Center for Data and Knowledge Integration for Health (CIDACS). These were licensed for exclusive use in the present study and, due to the privacy rules of the Brazilian Ethics Committee, are not openly available. Upon request with adequate justification and approval of an ethics committee, controlled access to data is evaluated; if possible, allowed access.

## Declaration of interests

We declare no competing interests.
