# Corrigendum to “Recent changes in growth trajectories: a population-based cohort study of over 5 million Brazilian children born between 2001 and 2014” [The Lancet Regional Health—Americas 2024; Volume 32, 100721, Published: March 27, 2024]

**DOI:** 10.1016/j.lana.2024.100787

**Published:** 2024-06-01

**Authors:** Carolina Santiago-Vieira, Gustavo Velasquez-Melendez, Rita de Cássia Ribeiro Silva, Elizabete de Jesus Pinto, Maurício L. Barreto, Leah Li

**Affiliations:** a School of Nursing, Universidade Federal de Minas Gerais, Belo Horizonte, Brazil; b School of Nutrition, Federal University of Bahia, Salvador, Brazil; c Center of Data and Knowledge Integration for Health (CIDACS), Fiocruz Bahia, Salvador, Brazil; d Health Sciences Center, Federal University of Recôncavo da Bahia, Santo Antônio de Jesus, Brazil; e Population, Policy and Practice Research and Teaching Department, Great Ormond Street Institute of Child Health, University College London, London, UK

**The authors wish to correct:**

1. In the methods section, under data analysis, in the sentence that reads: “The best-fitting two-degree fractional polynomials were the same for both cohorts, and for height trajectories included age and age3 for both sexes”, the square root sign for the age term is missing. Therefore, the sentence should read: “The best-fitting two-degree fractional polynomials were the same for both cohorts, and for height trajectories included √age and age3 for both sexes.”
2. In the results, in Table 1, in the height-for-age index (z-score), the values for Cohort 2 (2008–2014) should be changed. For boys, the height-for-age (z-score) from −0.339 to **−0.049**, and for girls, the height-for-age (z-score) from −0.267 to **−0.029**.

It is important to note that these corrections stem from typographical errors and do not affect the overall findings, interpretations, or conclusions of the study.

