# Supplementary material for: Recent changes in growth trajectories: a population-based cohort study of over 5 million Brazilian children born between 2001 and 2014
Source: Lancet Reg Health Am. 2024 Mar 27;32:100721. doi: 10.1016/j.lana.2024.100721 (PMC11019368; doi:10.1016/j.lana.2024.100721)

**Supplementary materials**

**Table S1**. Mean height (cm) and 95% confidence interval (CI) by age for boys and girls in the first (2001-07) and second (2008-14) cohorts and the difference by cohort estimated using mixed effect models with fractional polynomials (N= 5,750,242)

| Age (years) | 2001-07 |  | 2008-14 |  |  |
| --- | --- | --- | --- | --- | --- |
| Boys | Mean | 95% CI | Mean | 95% CI | Change (cm)* |
| 3 | 91.7 | (91.7, 91.8) | 92.8 | (92.7, 92.9) | 1.1 |
| 4 | 100.0 | (99.9, 100.0) | 101.0 | (100.9, 101.1) | 1.0 |
| 5 | 107.2 | (107.1, 107.3) | 108.2 | (108.1, 108.3) | 1.0 |
| 6 | 113.7 | (113.6, 113.8) | 114.8 | (114.6, 114.9) | 1.1 |
| 7 | 119.8 | (119.7, 112.0) | 120.9 | (120.7, 121.0) | 1.1 |
| 8 | 125.6 | (125.5, 125.8) | 126.6 | (126.5, 126.8) | 1.0 |
| 9 | 131.1 | (131.0, 131.3) | 132.1 | (132.0, 132.3) | 1.0 |
| 10 | 136.4 | (136.3, 136.6) | 137.5 | (137.3, 137.7) | 1.1 |
| Girls |  |  |  |  |  |
| 3 | 91.0 | (90.9, 91.0) | 92.0 | (91.9, 92.1) | 1.0 |
| 4 | 99.1 | ( 99.0, 99.2) | 100.2 | (100.1, 100.3) | 1.1 |
| 5 | 106.4 | (106.3, 106.5) | 107.4 | (107.3, 107.5) | 1.0 |
| 6 | 113.0 | (112.9, 113.1) | 114.1 | (114.0, 114.2) | 1.1 |
| 7 | 119.3 | (119.2, 119.4) | 120.3 | (120.2, 120.5) | 1.0 |
| 8 | 125.3 | (125.2, 125.4) | 126.3 | (126.2, 126.5) | 1.0 |
| 9 | 131.1 | (131.0, 131.3) | 132.2 | (132.1, 132.4) | 1.1 |
| 10 | 136.9 | (136.8, 137.1) | 138.01 | (137.8, 138.2) | 1.1 |

*p<0.05 for change in height between cohorts

**Table S2**. Mean BMI (kg/m^2^) and 95% confidence interval (CI) by age for boys and girls in the first (2001-07) and second (2008-14) cohorts and the difference by cohort estimated using mixed effect models with fractional polynomials (n= 5,750,214)

| Age (years) | 2001-07 |  | 2008-14 |  |  |
| --- | --- | --- | --- | --- | --- |
| Boys | Mean | 95% CI | Mean | 95% CI | Change (kg/m^2^)* |
| 3 | 16.16 | (16.13, 16.19) | 16.22 | (16.18, 16.25) | 0.06 |
| 4 | 15.84 | (15.80, 15.89) | 15.90 | (15.86, 15.95) | 0.06 |
| 5 | 15.72 | (15.66, 15.77) | 15.77 | (15.72, 15.83) | 0.05 |
| 6 | 15.77 | (15.72, 15.83) | 15.83 | (15.77, 15.89) | 0.06 |
| 7 | 16.01 | (15.95, 16.09) | 16.07 | (16.00, 16.15) | 0.06 |
| 8 | 16.45 | (16.37, 16.53) | 16.50 | (16.42, 16.59) | 0.05 |
| 9 | 17.07 | (16.97, 17.16) | 17.12 | (17.03, 17.22) | 0.05 |
| 10 | 17.87 | (17.76, 17.97) | 17.92 | (17.82, 18.03) | 0.05 |
| Girls |  |  |  |  |  |
| 3 | 15.99 | (15.86, 16.12) | 16.07 | (15.94, 16.21) | 0.08 |
| 4 | 15.63 | (15.46, 15.81) | 15.71 | (15.54, 15.89) | 0.08 |
| 5 | 15.55 | (15.34, 15.77) | 15.63 | (15.41, 15.84) | 0.08 |
| 6 | 15.69 | (15.44, 15.95) | 15.77 | (15.51, 16.03) | 0.08 |
| 7 | 16.01 | (15.72, 16.31) | 16.09 | (15.79, 16.39) | 0.08 |
| 8 | 16.49 | (16.15, 16.84) | 16.57 | (16.22, 16.91) | 0.08 |
| 9 | 17.11 | (16.73, 17.50) | 17.19 | (16.80, 17.58) | 0.08 |
| 10 | 17.85 | (17.42, 18.28) | 17.93 | (17.49, 18.36) | 0.08 |

*p<0.05 for change in BMI between cohorts

**Figure S1: Observed and estimated mean trajectory of height (cm), at different ages for the 2001-07 and 2008-14 cohorts (N= 5,750,242).**

**
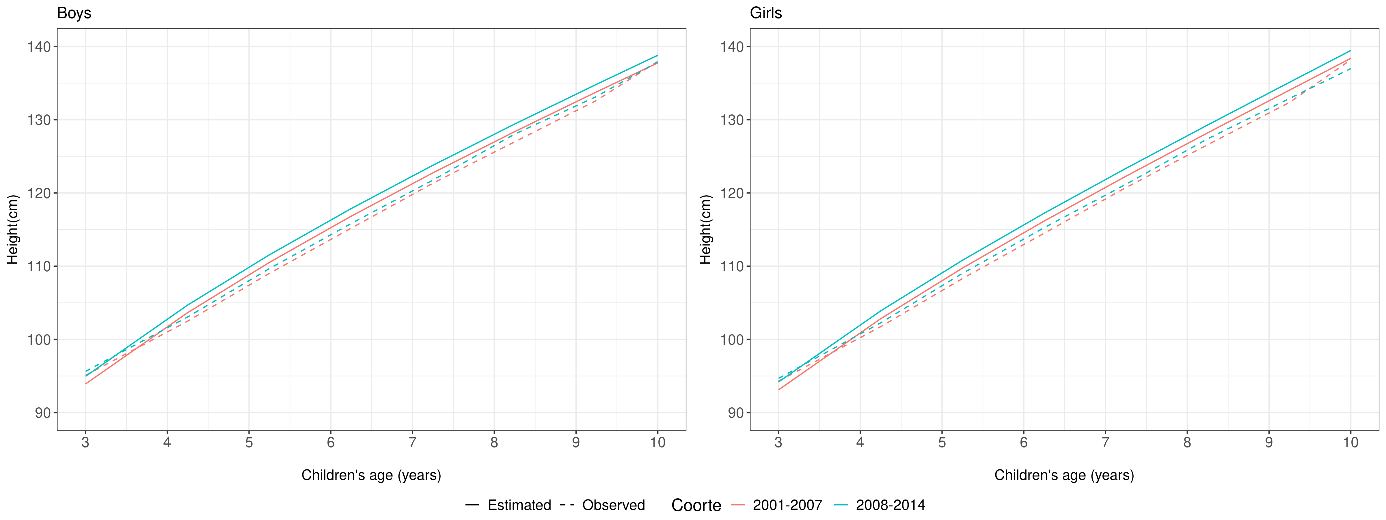
**

**Figure S2: Observed and estimated mean trajectory of BMI (kg/m^2^), at different ages for the 2001-07 and 2008-14 cohorts (N= 5,750,242).**


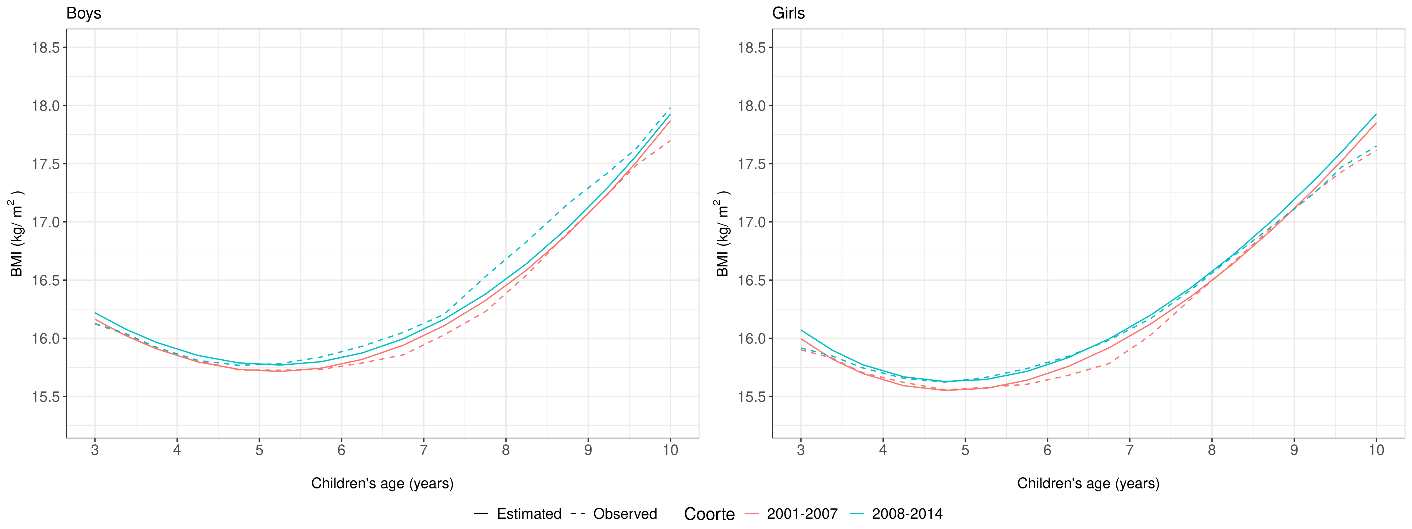

Supplement: Supplementary Figs. S1 and S2 and Tables S1 and S2 [file mmc1.docx]
